# Host-design strategies of zinc anodes for aqueous zinc-ion batteries

**DOI:** 10.1039/d4ra04353g

**Published:** 2024-07-22

**Authors:** Xuanyu Zhou, Tingting Ruan, Jie Xu, Chenhao Li, Shixuan Huang, Jianping Zhou, Shengli Lu, Rensheng Song, Ruhong Li

**Affiliations:** a School of Biological and Chemical Engineering, Zhejiang University of Science and Technology Hangzhou 310023 China 121074@zust.edu.cn jpzhou@zust.edu.cn lushengli@zust.edu.cn; b College of Environmental and Chemical Engineering, Dalian University Dalian 116622 China songrensheng@dlu.edu.cn; c ZJU-Hangzhou Global Scientific and Technological Innovation Center, Zhejiang University Hangzhou 311215 China

## Abstract

Aqueous zinc ion batteries (AZIBs) have garnered considerable interest as an eco-friendly, safe, and cost-effective energy storage solution. Although significant strides have been made in recent years, there remain technical hurdles to overcome. Herein, this review summarizes in detail the primary challenges confronting aqueous zinc ion batteries, including the rampant dendrite growth, and water-induced parasitic reactions, and proposes host-engineering modification strategies focusing on optimizing the structure design of the zinc anode substrates, involving three-dimensional structure design, zincophilicity regulation, and epitaxial-oriented modification, and comprehensively analyzes the structure–activity relationship between different modification strategies and battery performance. In addition, we highlight the research trends and prospects in future anode modification for aqueous zinc-ion batteries. This work offers valuable insights into advanced Zn anode constructions for further applications in high-performance AZIBs.

## Abstract 2

Host-design optimization strategies for zinc anode are systematically summarized. Reasonable structural regulation can control interfacial mass transfer, uniform zinc nucleation, and regulate crystal growth, thereby stabilizing the zinc anode.

## Graphical abstract

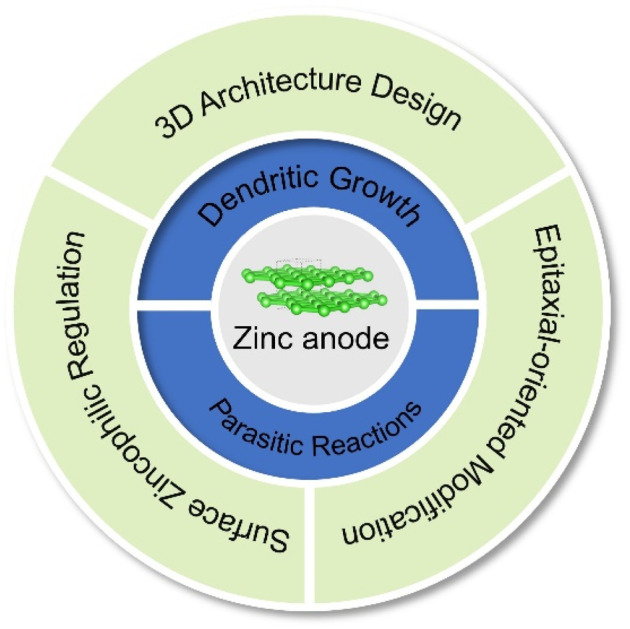

## 1. Introduction

The imminent crisis of ecological environment degradation and depletion of non-renewable energy sources dominated by fossil fuels have promoted the prosperous development of clean new energy technologies such as solar energy, wind energy, water energy, and biomass energy worldwide.^1–3^ With increasing demands on energy storage technology, battery energy storage implementation represents a promising solution that simultaneously resolves intermittency and fluctuation issues of renewable energy. With support from battery systems, the utilization of renewables could be prominently enhanced to store excess power from unstable energy sources like solar and wind, and break through the limitations of practical application scenarios presented by climate, time, and geographical conditions, thereby curbing carbon emissions, and propelling the development of energy systems towards a cleaner, more efficient, and sustainable trajectory.^4–7^

Among the multitude of batteries currently in use, lithium-ion batteries have gained widespread application in portable electronics and vehicles owing to their extended cycle life, high energy density, and enhanced stability.^8^ However, the advancement of lithium-ion batteries has been impeded by potential safety risks and the scarcity of lithium resources, particularly the susceptibility to fire and explosion when subjected to improper conditions such as overcharging, high temperatures, or physical damage.^9,10^ In contrast, aqueous zinc-ion batteries (AZIBs) stand out for their appealing merits of high energy and long circular life since zinc metal anode exhibits high theoretical specific capacity (volumetric capacity of 5855 mA h cm^−3^, mass-specific capacity of 820 mA h g^−1^) and low redox potential (−0.76 V relative to the standard hydrogen electrode).^11–13^ Additionally, zinc possesses abundant resources, high safety without suffering from the hazards experienced by metallic lithium such as combustion and explosion, and favorable recyclability for easy reuse.^14,15^ Leveraging these advantages, AZIBs, an appealing battery chemistry, have received extensive interest and made substantial progress.^16^ However, the issues of unavoidable dendrite growth and solvent-induced parasitic reactions such as corrosion, passivation, and gas evolution cause reduced reversibility of the zinc anode and even battery failure, ultimately making it unfeasible for further applications in aqueous zinc-ion batteries.^17^ To address these problems, various strategies have been proposed from various aspects to enhance the stability of the zinc anode, such as interfacial coating modification, electrolyte material innovation, and functionalized membrane design, which have effectively mitigated dendrite growth and suppressed side reactions, thereby significantly improving the cycle lifespan of AZIBs.^18–22^

This study delves into the reaction kinetics of zinc ion deposition and dissolution and intrinsic mechanisms of dendrite growth and other parasitic reactions, which are the theoretical basis for the modification of the anode structures. With the focus on the structural design of the anode hosts, we comprehensively discuss the internal relation of the substrate surface condition and the homogeneity of Zn deposition and elaborate the design principles of three modification strategies of anode substrates, including three-dimensional structure design, zincophilicity regulation, and epitaxial-oriented modification to achieve high-performance zinc anode. Finally, prospects are presented according to zinc substrate engineering strategies that help uniform deposition of Zn^2+^ ions and protect the zinc anodes against the aqueous electrolytes, thereby enabling reliable reversibility and cycle life of the zinc anodes.

## 2. Challenges of zinc anodes and modification strategies

**Fig. 1:**
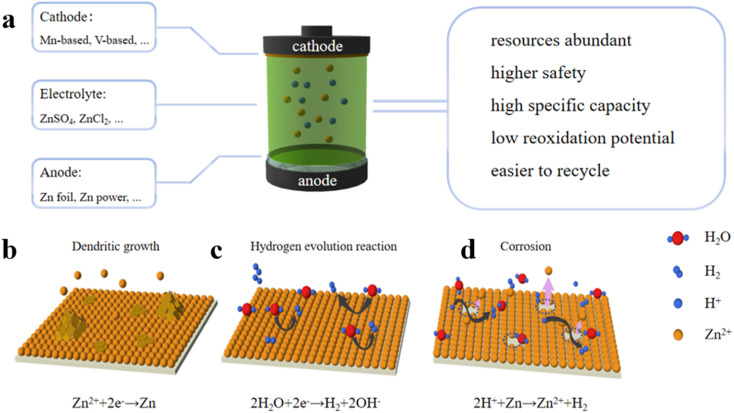
(a) Schematic diagram of AZIBs. Challenges of zinc anodes: (b) dendritic growth, (c) hydrogen evolution, and (d) parasitic corrosion reaction.

As the battery industries undergo heavy development, rechargeable aqueous zinc-ion batteries have aroused booming attention. The use of zinc metals as anode materials traces back about 200 years to the introduction of alkaline zinc–manganese batteries. Notably, in 2012, Xu *et al.* discovered that zinc ions in neutral or weakly acidic electrolytes could undergo reversible embedding and removal reactions in the MnO_2_ cathodes (Fig. 1a).^23^ Considering the moderate reactivity of zinc with water, metallic zinc emerged as a promising anode candidate for the development of water-based zinc-ion batteries. However, the direct use of zinc metal as an anode is still limited by its physical and chemical instability during the charging and discharging processes, involving issues such as dendrite growth (Fig. 1b), hydrogen precipitation (Fig. 1c), corrosion and passivation (Fig. 1d). These challenges significantly impede the advancement of aqueous zinc-ion batteries. Therefore, it is of great significance to develop regulating ideas for Zn anodes to inhibit zinc dendrite growth and mitigate side reactions for expediting the commercialization of AZIBs.

### 2.1 Growth of zinc metal dendrites

The growth of metallic zinc dendrites represents a major hurdle in AZIBs, which mainly occurs during the charging processes. When being electrochemically cycling, the mass transfer of zinc ions in the water-based electrolyte is affected by multiple factors such as electric field distribution and anode surface texture, which causes zinc ions to be more likely to nucleate at low-energy sites and reduce to small protrusions of metallic zinc on the surface of the anode substrates. Due to the strong electric field distribution generated at the protrusion, the tip effect is amplified at the initial deposition sites with the continuous deposition of zinc, resulting in more uneven distribution of ion concentration and electric field, ultimately leading to the formation of dendrites.^24,25^ As dendrites grow, they increase the contact area between the anode and electrolyte, causing newly exposed zinc to continuously react with the electrolyte. This ongoing reaction intensifies the consumption of the electrolyte, thereby reducing the coulombic efficiency of the overall battery and even causing battery failure. At the same time, when the dendrites on the electrode surface develop sharply to a certain extent, they can not only pierce the separator leading to a short circuit in the battery, but the dendrites that fall off form “dead zinc”, which would reduce the battery capacity and increase the internal resistance, causing the battery performance to decline sharply, especially at high current densities, the battery will rapidly fail. Therefore, addressing the growth of metallic zinc dendrites is crucial to enhancing the stability and lifespan of aqueous zinc ion batteries.^26^

### 2.2 Water-induced parasitic reactions

In addition to the zinc dendrite issue, the hydrogen evolution reaction (HER) also competes with zinc deposition at the end of charging, despite relatively high HER overpotential of zinc anode. Especially in acidic electrolytes, the zinc metal anode is unstable and will continue to consume H^+^ ions and irreversibly produce H_2_ gas to escape, causing the battery to crack or other safety hazards.^27–29^ As the HER proceeds, the pH values of the local electrolyte environment increase, inevitably leading to the formation of Zn(OH)_4_^2−^ chemicals on the surface of the zinc anode during the discharging stage, which subsequently decompose into insulating ZnO by-products, resulting in the coverage of the passivation layer.^30–32^ The formation of the passivation layer reduces the active zinc surface area, impeding direct contact between zinc metal and electrolyte, thus reducing the utilization of the zinc anode and even terminating the discharge process. In neutral or weakly acidic electrolytes, taking ZnSO_4_ aqueous electrolyte as an example, zinc ions react with SO_4_^2−^ and H_2_O to generate by-products (Zn_4_SO_4_(OH)_6_*x*H_2_O), which are randomly stacked to form a loose interface layer, thus slowing electron/mass transmission and increasing interface impedance of zinc electrodes.^33–36^ Furthermore, due to the loose structure, zincate by-products may peel off during cycling, causing the electrode surface to be re-exposed to electrolytes and increasing corrosion risk.

Owing to the thermodynamic instability of zinc metals described by Pourbaix diagram,^37^ the chemical corrosion reactions occur spontaneously originating from redox reactions between the zinc anode and the aqueous solution, which will be exacerbated by electrochemical corrosion through the formation of galvanic corrosion microcells, thus further reducing the utilization of zinc electrode. In addition, Zn metal corrosion, especially electrochemical corrosion, is accompanied by hydrogen evolution, resulting in local enhancement of OH^−^ concentration and pH, aggravating the formation of passivation products at the interface.^38^

### 2.3 Modification strategies towards stable Zn anode

In general, these undesired reactions are not independent of each other, as the growth of zinc dendrites increases the contact area between the electrode and the electrolyte, thereby providing a greater number of reaction sites and accelerating HER behavior. The accumulation of OH^−^ anions caused by HER will form electrochemically inert passivation layers at the anode interface, resulting in rough surfaces that increase electrode impedance and polarization, and in turn, aggravate the formation of dendrites. The aforementioned side reactions have a detrimental impact on the output energy density and the actual service life of zinc anodes, thereby reducing the viability of zinc anodes in practical energy storage systems. Given the correlation between these problems and their inherent mechanisms, researchers have proposed various strategies based on the optimization of the electrolyte and structural design of the zinc anode substrate to comprehensively solve these issues, thereby improving the performance and cycle life of AZIBs and promoting their development in the field of energy storage technology.

Electrolyte optimization has a significant impact on the electrochemical performance of zinc anode. Table 1 lists the engineering strategies and corresponding physicochemical characteristics of aqueous electrolytes. In common dilute electrolytes, one Zn^2+^ is believed to form a solvation sheath structure with nearly six water molecules, *i.e.* Zn(H_2_O)_6_^2+^. The water-rich solvation structure can lead to side reactions of water at the interface, resulting in irreversibility of the Zn anode. Concentrated electrolytes were developed to overcome the issues of zinc anode. The water molecules in concentrated electrolytes are mainly confined in Zn^2+^ ion-solvated shells. Thus, the electrochemical stable window of the electrolyte is largely extended and the water activities are strongly limited, achieving the suppression of HER.^41^

**Table 1:** Characteristics of various electrolytes used in AZIB

Electrolyte	Formulation	Viscosity	Conductivity (S cm^−1^)	Electrochemical window	Cost	Ref
Dilute	2 M ZnSO_4_	Low (∼10^−3^)	High (∼10^−2^ to 10^−1^)	Narrow	Low	39
Concentrated “water-in-salt”	30 M ZnCl_2_	High (∼10^−1^)	Low (∼10^−3^)	Wide	High	40
Localized concentrated	30 M ZnCl_2_-TFE	Medium (10^−2^)	Medium (10^−3^ to 10^−2^)	Wide	High	41
Additive	ZnCl_2_–H_2_O–DMSO	Medium (10^−2^)	Medium (10^−3^ to 10^−2^)	Medium	Low	42

In addition to changing the zinc salt concentration, recent studies have used functional additives to address the problem in AZIB. The mechanisms by which additives improve electrochemical stability can be divided into three categories.^39^ First, absorbtion-type additives tend to adsorb on the surface of the zinc anode to isolate water and may drive Zn^2+^ to deposit closely in adjacent plane areas, ultimately inducing Zn^2+^ deposition uniform. In addition, since electrolyte additives occupy the active sites formed by hydrogen adsorption on the surface of the zinc anode, HER and corrosion can be effectively suppressed. Second, the introduction of self-sacrificial additives into aqueous electrolytes can construct an adaptive solid electrolyte interface *in situ* during the cycling process. The formation of the interface can effectively passivate the electrode, inhibit electrolyte side reactions, and homogenize the ion flux. Third, the introduced additive (also called co-solvent) can destroy the solvated structure of Zn^2+^ and form a solvation sheath in which the co-solvent participates in coordination, lowering the water molecule content in the solvent sheath structure. In addition, various additives contain O and N elements with strong electronegativity, which can form hydrogen bonds with water molecules in the electrolyte, thereby destroying the hydrogen bond network established by free water. Currently, electrolytes have made great progress in avoiding water-induced side reactions and stabilizing Zn anodes.^37^ However, during the deposition of zinc anode, the Zn^2+^ ions in electrolytes reduce to Zn^0^ atom, gather into Zn nuclei in the nanoscale, grows continuously, and finally form to crystallized Zn metals on the microscale. As the place where the deposition reaction takes place, the zinc anode host can also greatly affect the electrochemical behavior of the Zn anode.

## 3. Structural design of zinc anodes

Pure zinc metal adopts a hexagonal close-packed (HCP) structure (space group: *P*6_3_/*mmc*), with the (002) plane representing the close-packed face characterized by the lowest surface energy of 0.02 eV Å^−1^. Consequently, zinc deposits tend to exhibit a preferential orientation, grow along the *ab* plane, and deposit as the hexagonal platelets. However, the actual morphology of zinc deposits is influenced by crystal thermodynamics and dynamics, which may cause variations in deposition. The geometrical features of electrochemically deposited zinc primarily hinge on the zinc nucleation and growth processes. These processes are theoretically controlled by zincophilicity of the substrates and the local current or electric field distribution around the substrates. Notably, the former presents the ability to bond Zn, which is highly related to the surface chemical and physical conditions, while the latter is closely tied to the structure of substrates. To optimize the deposition behavior of zinc anodes, rational substrate design interventions are crucial to regulate the nucleation and growth processes of Zn on the substrate. From the perspective of zinc anode substrate modification, this paper sums up the recent research progress on three modification strategies, including three-dimensional structural design, zincophilicity regulation, and epitaxial-oriented modification, which may offer directions for future research of advanced zinc anodes and help to accelerate their practical applications in AZIBs.

### 3.1 Three-dimensional structure design for uniform mass transfer

**Fig. 2:**
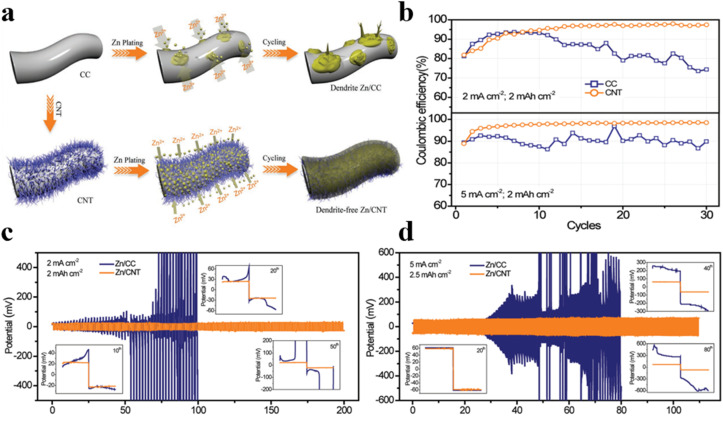
(a) Schematic drawing zinc deposition process for Zn/CC and Zn/CNT electrodes.^53^ (b) Coulombic efficiency comparison of pure CC and CNT anodes under 2 mA cm^−2^ and 5 mA cm^−2^.^53^ Voltage curves of symmetric cells assembled with Zn/CC and Zn/CNT anodes (c) at 2 mA cm^−2^ and 2 mA h cm^−2^, and (d) at 5 mA cm^−2^ and 2.5 mA h cm^−2^.^53^

The electric and/or ion concentration field surrounding the anode substrates plays a significant role in the nucleation and growth processes of metal deposits. The traditional substrates of zinc metal anode are planar, once small protrusions formed on the planar structure, the uniform electric field will be disturbed. The redistributed electric field becomes concentrated around the protrusions, leading to amplified deposition on them, eventually resulting in dendrite formation. In contrast, three-dimensional (3D) substrates with expanded specific surface areas can homogenize current density, effectively reducing it below the critical current density for dendrite growth during charging and discharging. Consequently, metal deposits preferentially form inside the skeleton rather than on the outer surface of 3D substrates, thereby inhibiting dendrite formation. Moreover, 3D substrates with precisely controlled nanopores can induce the formation of interface-localized concentrated electrolytes. The space charge effect of nanopores can modulate the charge density in the electric double layer (EDL), providing a localized concentrated electrolyte environment at the interface without any change in the bulk electrolyte. Concentrated electrolytes have proven effective in protecting zinc anodes due to the unique EDL with reduced free water and abundant zinc ions. Recent studies have demonstrated that 3D substrates could mitigate the capacity loss of zinc anodes during calendar aging. As compared to planar substrates, 3D hosts with the same zinc loading exhibit a smaller loss of coulombic efficiency. This enhancement is attributed to the significantly larger surface areas of 3D substrates, which can facilitate faster mass transfer and inhibit the formation of inactive zinc. In the realm of 3D structural anode modification, researchers have made some progress by using 3D hosts (such as porous carbon materials, MOF-based materials, and metal materials, *etc.*) to construct 3D porous zinc anode structures to accommodate zinc deposition and suppress dendrite growth, thus achieving enhanced cycling stability, as shown in Table 2.^27^ For instance, Zeng *et al.* employed highly conductive carbon nanotube (CNT) materials to construct three-dimensional frameworks *via* chemical vapor deposition (CVD) and obtained impurity-free Zn/CNT anodes after electrodeposition.^53^ 3D CNT frameworks could expand the specific area and reduce the nucleation overpotential, and are conducive to the uniform distribution of the electric field, ensuring that Zn^2+^ could be deposited more uniformly on the entire electrode surface, thereby effectively eliminating zinc dendrites or other defects brought by harmful by-products during charging and discharging processes and realizing highly reversible zinc deposition/stripping (Fig. 2a). Comparative electrochemical analysis between Zn/CC and Zn/CNT anodes in symmetric coin cells demonstrates the superior performance of the impurity-free Zn/CNT anode in terms of polarization voltage and cycling behaviors, underscoring the low polarization and high stability of porous carbon materials (Fig. 2b–d).

**Table 2:** Comparison of anode materials based on 3D structure design strategies

Anode material	Electrolyte	Voltage hysteresis	Cycling performance	Ref.
Zn foil and Cu foam (RCZ)	2 M ZnSO_4_	∼120 mV (1 mA cm^−2^)	1800 h (1 mA cm^−2^)	43
Carbon network NCL-Zn	2 M ZnSO_4_	71 mV (1 mA cm^−2^)	4000 h (1 mA cm^−2^)	44
Cu mesh	1 M ZnSO_4_	20 mV (1 mA cm^−2^)	340 h (1 mA cm^−2^)	45
Alloy network Cu@Cu_3_Zn	3 M Zn(CF_3_SO_3_)_2_	∼100 mV (1 mA cm^−2^)	1900 h (1 mA cm^−2^)	46
Expanded graphite Zn@Cu-Ps/EG	3 M Zn(CF_3_SO_3_)_2_	∼100 mV (10 mA cm^−2^)	3000 h (10 mA cm^−2^)	47
3D Zn@Cu-Sn@SSM	2 M ZnSO_4_	∼95 mV (10 mA cm^−2^)	1050 h (10 mA cm^−2^)	48
Nanofiber Zn@PBI-Cu	2 M ZnSO_4_ + 0.1 M MnSO_4_	70 mV (10 mA cm^−2^)	300 h (10 mA cm^−2^)	49
3D AgNPs@CC	1 M Zn(CF_3_SO_3_)_2_	∼56 mV (2 mA cm^−2^)	800 h (2 mA cm^−2^)	50
3D Zn@N-VG@CC	2 M ZnSO_4_	∼6.2 mV (0.5 mA cm^−2^)	150 h (0.5 mA cm^−2^)	51
3D concave Zn	2 M ZnSO_4_ + 0.2 M MnSO_4_	∼100 mV (2 mA cm^−2^)	1000 h (2 mA cm^−2^)	52

**Fig. 3:**
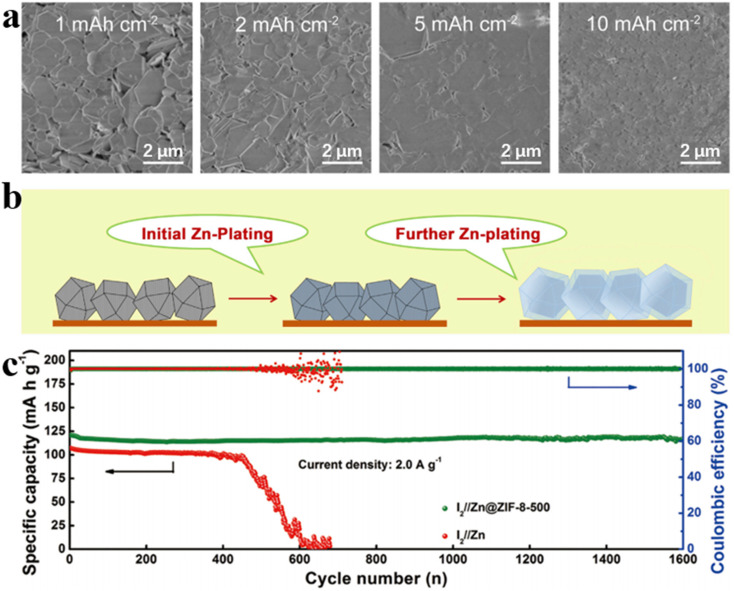
(a) SEM images of Zn deposits when cycled at a current density of 1 mA cm^−2^ for 1, 2, 5, and 10 h.^54^ (b) Schematic showing Zn deposition process.^54^ (c) Capacity retention and coulombic efficiency under a current density of 2 A g^−1^.^54^

With the development of 3D carbon material research, metal–organic framework (MOF) porous materials have garnered increasing attention from researchers. By optimizing the heat treatment temperature, Xia *et al.* demonstrated that ZIF-8 obtained at 500 °C exhibited optimal performance upon zinc deposition/exfoliation. The superiority is attributed to the trace amount of zinc existing in the ZIF-8 hosts, which can not only provide homogeneous nucleus sites for zinc deposition to facilitate uniform deposition across the substrate, but also limit water activity in the electrolyte due to high over-potential during hydrogen evolution, thus effectively reducing water-induced parasitic reactions (Fig. 3a and b).^54^ When assembled into I_2_//Zn@ZIF-8-500 cells, remarkable capacity retention of 97% and nearly 100% coulombic efficiency are achieved after 1600 electrochemical cycles at the current density of 2 A g^−1^ (Fig. 3c). This outcome presents a promising, cost-effective pathway for the development of highly reversible zinc anodes and lays the foundation for further advancement in the field of high-performance AZIBs.

**Fig. 4:**
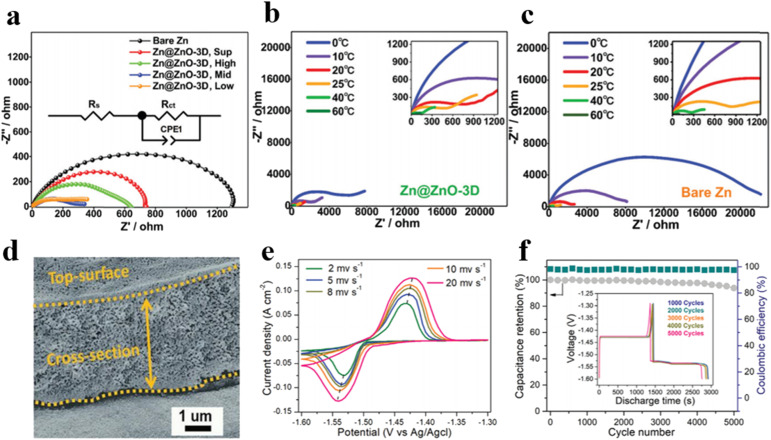
Nyquist plots of Zn@ZnO-3D symmetric cells of (a) Zn anodes at room temperature, (b) Zn@ZnO-3D anodes, and (c) bare Zn anode at different temperatures.^56^ (d) Cross-section SEM image of 3D Zn–Cu anode after cycling.^57^ (e) Cyclic voltammetry (CV) curves at different sweep rates.^57^ (f) Capacity retention of 3D Zn–Cu anode at 4 mA cm^−2^.^57^

In previous studies on 3D porous anodes constructed with carbon or MOF materials as functional hosts in AZIBs, researchers have predominantly focused on electrochemical capabilities and morphological characteristics, ignoring the analysis of zinc migration and deposition kinetics.^55^ It has been established that the sluggish kinetics behaviors of zinc diffusion would enlarge concentration polarization and hasten dendrite growth, resulting in diminished electrochemical performance. Therefore, it is imperative to optimize the transport process of zinc ions. Liang *et al.* reported a liquid-phase synthesis method to deposit three-dimensional nanoporous ZnO structures onto the surface of zinc anodes, and electrochemical impedance spectroscopy (EIS) was employed to assess the charge transfer resistance of the zinc anodes under various preparation conditions and different temperatures in symmetric cells (Fig. 4a–c).^56^ The results reveal the lower *R*_ct_ values Zn@ZnO-3D, indicating that the three-dimensional anode structures facilitate the reduction of local current densities thereby minimizing interfacial side reactions, and further confirming that the Zn@ZnO-3D is conducive to accelerated deposition kinetics of zinc ions.

The utilization of 3D metallic materials can also significantly enhance the deposition and stripping properties of zinc anodes. Liu *et al.* fabricated 3D Zn–Cu alloy anodes with an ordered porous structure *via* thermal and electrochemical treatments (Fig. 4d).^57^ In comparison to two-dimensional counterparts, 3D Zn–Cu anodes offer increased reaction interfaces and active sites owing to augmented contact surface areas, and the conductive channels facilitate the rapid transfer of ions and electrons, which helps to reduce the internal resistance and improve the energy efficiency of the cells. Remarkably, stable deposition/exfoliation is sustained for over 300 hours at 2 mA cm^−2^ with outstanding cycling performance (Fig. 4e and f). Moreover, compared with other metals, copper metal exhibits higher zincophilicity, thereby lowering the nucleation barrier for zinc deposition and further promoting uniform zinc ion deposition. Although optimized substrate design with 3D structures can modulate zinc stripping/plating processes and foster dendrite-free zinc anodes, other parasitic reactions like hydrogen evolution and aqueous corrosion persist due to the weakly acidic aqueous environment. Engineering the substrates alone cannot eliminate these reactions. To safeguard zinc anodes from aqueous electrolytes, extensive research is imperative, particularly on the interface construction for zinc anode protection.

### 3.2 Zincophilic regulation for homogeneous Zn nucleation

**Fig. 5:**
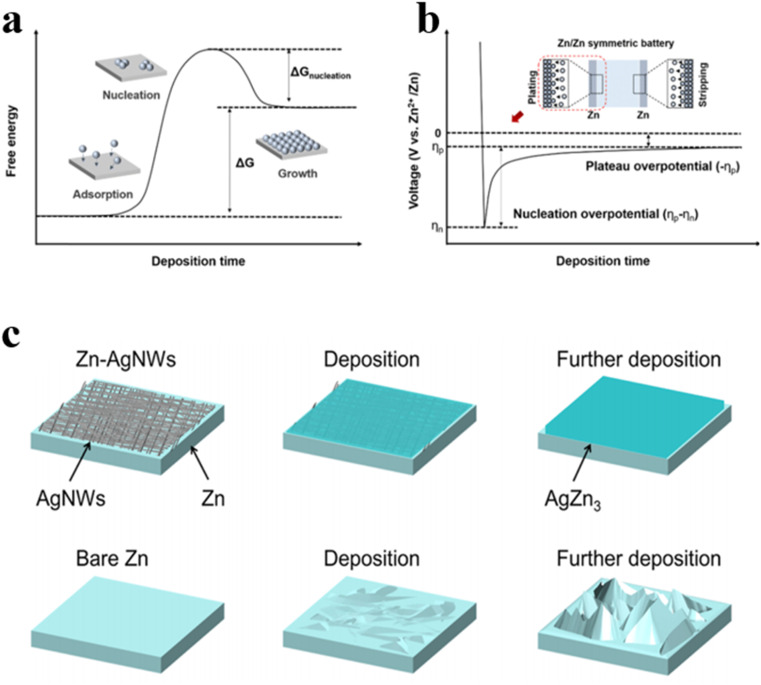
(a) The free energy graph *versus* deposition time.^58^ (b) Characteristic voltage diagram for Zn//Zn symmetric battery.^58^ (c) Schematic illustration of surface morphology evolutions of Zn-AgNWs and pure Zn metal anodes upon Zn deposition.^59^

Zinc deposition is triggered by heterogeneous nucleation, necessitating the overcoming of the nucleation energy barrier (Δ*G*_nucleation_) to form the solid phase. Δ*G*_nucleation_ electrochemically reflects the process of Zn deposition through the nucleation overpotential in the voltage distribution (Fig. 5a and b).^58^ Typically, voltage drops rapidly until the potential difference provides adequate driving force for Zn metal nucleation, with the lowest potential reached denoted as *η*_n_. Subsequently, voltage gradually increases to a stable level, forming a potential plateau (*η*_p_). Both Δ*G*_nucleation_ and nucleation overpotential are closely linked to zincophilicity since the substrate hosting metallic Zn acts as a non-homogeneous catalyst for Zn electrochemical deposition. Generally, a high zincophilicity reduces the energy barrier for Zn nucleation, thus favoring uniform nucleation. Uniform nuclei formation around the substrate promotes preferential crystal growth at the nuclei sites rather than at undetermined locations, which is conducive to the uniform growth of zinc deposits. The zinc affinity of the substrate can be quantified to a certain extent by the binding energy between the zinc atom and the substrate. The higher the binding energy, the better the zinc affinity of the substrate. Studies have found that alloying modification of zinc anodes with the introduction of highly conductive metals like aluminum, copper, or silver can enhance zincophilic nature, thus improving anode conductivity and cycle stability. Li's group synthesized silver nanowires with excellent electrical conductivity and stability as coating layers for zinc anodes *via* the hydrothermal method.^59^ Due to the high zincophilicity, zinc deposition on the surface of silver nanowires (AgNWs) layers during charging and discharging drives the *in situ* formation of AgZn_3_ alloys. Since AgZn_3_ has a higher binding energy with zinc atoms, the redox potential of Zn^2+^/Zn_*x*_Ag_1−*x*_ pair is higher than that of Zn^2+^/Zn, favoring preferential Zn^2+^ deposition on the silver surface, which reduces nucleation behavior on the zinc anode surface and effectively controls the zinc deposition process (Fig. 5c). When cycled at a current density of 0.6 A g^−1^, the MnO_2_//Zn-AgNWs cells remain a capacity retention of 65% after 800 cycles. Consequently, constructing high-performance Zn-AgNWs holds vast potential for applications in AZIBs.

**Fig. 6:**
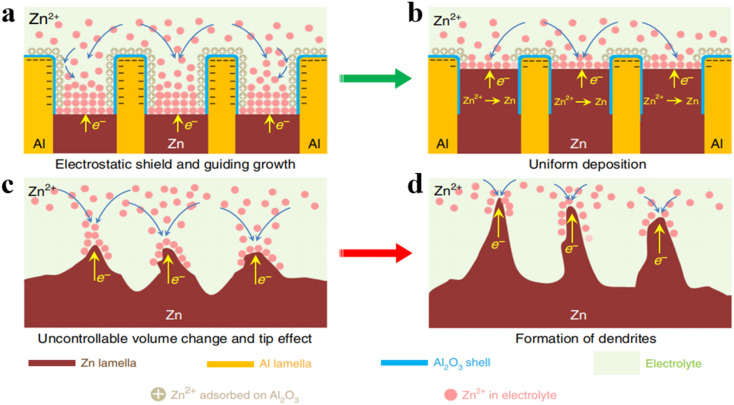
(a and b) Eutectic Zn/Al alloy with insulating Al_2_O_3_ shielding beneficial to the homogeneous deposition of Zn.^60^ Schematic diagram of the eutectic protocol to restrain Zn dendrites and cracks: (c and d) Undesired volume expansion and tip effect initiate Zn dendrite growth.^60^

Pre-designing the 3D structure of Zn anode is a common strategy. Recently, an approach to realize dendrite-free reversible anodes has been adopted by using the metallurgical approach to construct an alternating lamellar nanostructure of zinc and aluminum.^60^ The anode structure comprises a eutectic phase of zinc and aluminum elements, with the aluminum component acting as the framework for zinc deposition and the zinc component serving as the carrier for zinc ions, both of which form relatively small and uniformly distributed grain sizes with good strength and plasticity. Within the eutectic region, the formation of alumina insulating layers on the aluminum surface effectively prevents electron transfer between aluminum and zinc, which induces uniform zinc deposition between the layers (Fig. 6a, and b), while the pure zinc electrode exhibits numerous defects and dendritic formations (Fig. 6c and b). Even after 2000 cycles at 0.5 mA cm^−2^, the eutectic zinc–aluminum alloy anode remains smooth surfaces, effectively inhibiting zinc dendrite formation and growth.

**Fig. 7:**
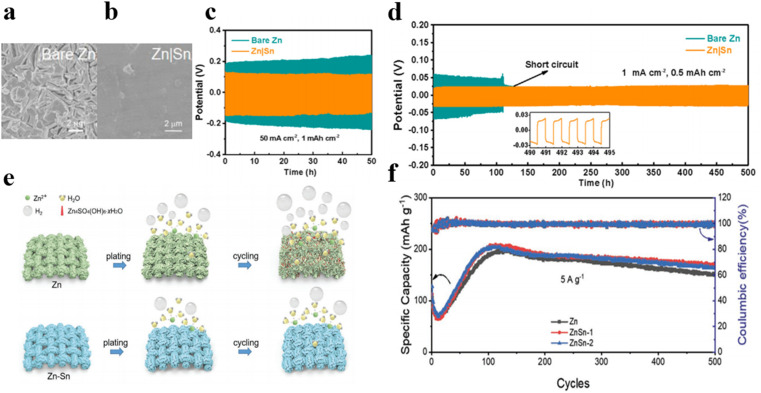
SEM images of (a) pure Zn electrode and (b) Zn|Sn anode in symmetric cell after 20 cycles at 1 mA cm^−2^ and 1 mA h cm^−2^.^62^ Cycling performance of bare Zn and Zn|Sn anodes (c) at 50 mA cm^−2^ and 1 mA h cm^−2^ and (d) at 1 mA cm^−2^ and 0.5 mA h cm^−2^.^62^ (e) Schematic graph of hydrogen evolution side-reaction on the Zn|Sn alloy electrode and pure Zn electrode.^63^ (f) Cycling capability of Zn‖V_2_O_5_ and ZnSn‖V_2_O_5_ batteries at 5 A g^−1^.^63^

It is worth noting that galvanic coupling corrosion occurs at bimetallic contacts due to the different corrosion potentials of the metals.^61^ To effectively prevent corrosion, corrosion-resistant tin coatings with a high Zn retention rate can be employed. Guo *et al.* constructed a uniform and sturdy Sn coating on zinc anodes by using the CVD method, which increases zinc nucleation sites, leading to uniform zinc deposition and exfoliation.^62^ Additionally, the Sn coating increases hydrogen evolution overpotential, effectively suppressing gas evolution reaction and corrosion behavior on the anode, thereby significantly enhancing zinc anode reversibility. The symmetrical battery assembled using Zn|Sn displays outstanding rate capability and durable cycling stability, and even operates for 50 hours under an ultra-high current density of 50 mA cm^−2^ (Fig. 7a–d). Furthermore, the nucleation overpotential of the zinc anode can be effectively reduced through alloying treatment.^58^ Wang's group fabricated zinc–tin alloy anodes *via* electrodeposition to inhibit hydrogen evolution reaction and dendrite growth (Fig. 7e).^63^ By monitoring hydrogen produced during charge and discharge cycles, the results quantitatively prove that the hydrogen production of zinc–tin alloyed electrodes is only half that of pure zinc electrodes. Moreover, Zn–Sn alloying helps to lower the zinc nucleation energy barrier, promoting uniform zinc deposition. In ZnSn-1‖V_2_O_5_ cells, the capacity remains 170.4 mA h g^−1^ after 500 cycles at 5 A g^−1^ with coulombic efficiency of about 99.8% (Fig. 7f). Zincophilic regulation by well-confining Zn nucleation sites can guarantee the uniform charge distribution and provide homogeneous nucleation sites, enabling uniform Zn stripping/plating while suppressing hydrogen generation and surface corrosion thereby effectively improving the cycle stability and extending the service life of the battery, as presented in Table 3.

**Table 3:** Comparison of anode materials based on zincophilic regulation strategies

Anode material	Electrolyte	Voltage hysteresis	Cycling performance	Ref.
MB@Zn	2 M ZnSO_4_	28.1 mV (0.2 mA cm^−2^)	1600 h (0.2 mA cm^−2^)	64
Bi@Zn	2 M ZnSO_4_	55 mV (10 mA cm^−2^)	2000 h (10 mA cm^−2^)	65
g-C_3_N_4_@Zn	2 M ZnSO_4_	∼60 mV (0.2 mA cm^−2^)	2900 h (0.2 mA cm^−2^)	66
Zn_*x*_Cu_*y*_/Zn	1 M Zn(OTF)_2_	∼65 mV (0.5 mA cm^−2^)	1900 h (0.5 mA cm^−2^)	67
Zn–Ti	3 M ZnSO_4_	27 mV (1 mA cm^−2^)	1100 h (2 mA cm^−2^)	68
Cu/Zn	3 M ZnSO_4_	∼50 mV (1 mA cm^−2^)	648 h (1 mA cm^−2^)	69
Zn@ZnS	2 M ZnSO_4_	43.6 mV (1 mA cm^−2^)	3000 h (1 mA cm^−2^)	70
NVPC@Zn	2 M ZnSO_4_	41 mV (2 mA cm^−2^)	1800 h (2 mA cm^−2^)	71
Zn@PA-ZnAl	2 M ZnSO_4_	∼80 mV (5 mA cm^−2^)	650 h (5 mA cm^−2^)	72
ZnCo	3 M ZnSO_4_	∼100 mV (5 mA cm^−2^)	620 h (5 mA cm^−2^)	73

### 3.3 Epitaxial-oriented modification to regulate Zn growth

**Fig. 8:**
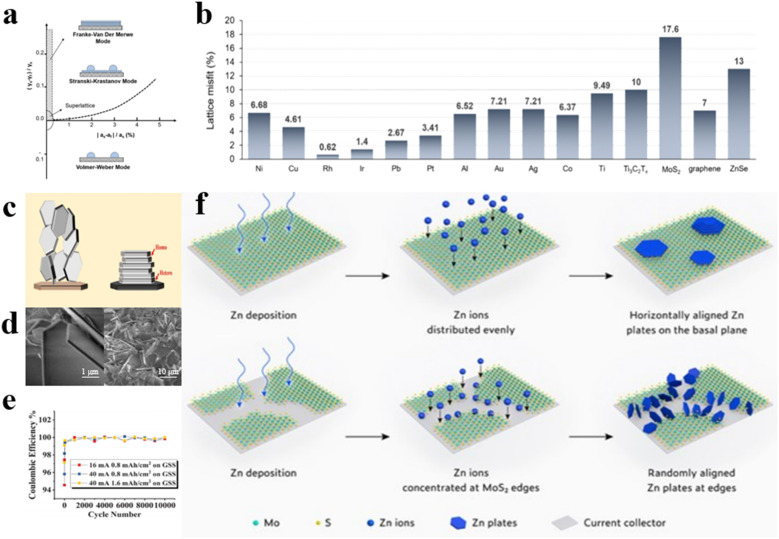
(a) Domains of three nucleation patterns as a function of lattice mismatch (*x*-axis) and surface energy differences (*y*-axis).^58^ (b) The lattice misfit fractions of Zn and other substrates.^58^ Schematic diagram (c) and SEM image (d) of epitaxial Zn electrodeposition.^74^ (e) coulombic efficiency of GSS anodes.^74^ (f) Zn deposition on MoS_2_ substrate verifying the design principle of epitaxial modification.^75^

The physicochemical characteristics of the substrates, particularly the degree of lattice mismatch between Zn crystals and the substrates, directly influence zinc deposition behavior. Theoretical models classify film growth into three modes: Frank–Vander Merwe, Volmer–Webber, and Stranski–Krastanov, distinguished by lattice mismatch and surface energy differences (determined by surface energy), as shown in Fig. 8a.^58^ Notably, homogeneous film formation will follow the Frank–Vander Merwe mode when lattice mismatch is minimal and surface energy difference is positive. Otherwise, deposits form isolated islands (following the Volmer–Webber mode) or combinations of films and islands (following the Stranski–Krastanov mode). Based on the deposition mechanisms, electrochemical deposition of metals can be modulated at the atomic level *via* epitaxial growth at the phase lattice interfaces on low lattice mismatch substrates, thus yielding regular and flat metal deposition morphologies (Fig. 8b). Archer's team demonstrated that the application of graphene as a substrate can induce the epitaxial growth mode of zinc anodes, thus defining the concept of epitaxially oriented modification (Fig. 8c).^74^ Unlike the modification methods that inhibit dendrite growth through inducing homogeneous nucleation, graphene host prompts epitaxial zinc deposition due to its highly matched lattice structure with zinc, contributing to the formation of stable zinc crystallization atop graphene (Fig. 8d). Upon electrodeposition, zinc metals will nucleate and grow along graphene crystal planes, forming an epitaxial layer with matching orientation of the graphene substrate, inhibiting dendrite formation. Since controlling the oriented arrangement of graphene flakes in a macroscopic substrate is beneficial to improving the crystallization properties of graphene, thereby improving the performance of the overall battery, researchers designed a scraper device. The sheer force of the scraper effectively induces the rearrangement of the disordered dispersed two-dimensional graphene sheets, resulting in the formation of an oriented aligned graphene film substrate. The zinc anode demonstrates exceptional reversibility, achieving a remarkable 99.9% coulombic efficiency with over 10 000 cycles at high currents of 16 and 40 mA (Fig. 8e).

In epitaxial-oriented modification studies, the choice of suitable substrate materials is essential. Precise control over zinc metal deposition is achievable through the design and optimization of appropriate substrate materials, thereby enhancing the energy density and cycle life of the battery. However, multi-oriented zinc metal deposition tends to occur on most substrate materials, sparking debate over the epitaxial relationship between substrate material and Zn metal. Wang *et al.* prepared 2D MoS_2_ materials with large areas of continuous mono-orientation as anode substrates, which can facilitate heterogeneous epitaxial crystallization and growth of Zn due to the low lattice mismatch and offer high homogeneity and smoothness over expansive areas (Fig. 8f).^75^ Once Zn deposits completely cover the substrate, subsequent Zn^2+^ deposition follows epitaxial growth behavior, ensuring uniform zinc deposition. By encasing the edges of single-oriented MoS_2_ substrates to eliminate possible competing reactions for zinc deposition on the edges and surfaces of the substrates, they obtained Zn films with nearly all (002) crystallographic orientations. Polar plot analysis results verify the epitaxial Zn growth on MoS_2_ substrates and confirm the effective suppression of Zn metal dendrite formation by using large-area, mono-oriented substrates with akin lattice structures.

**Fig. 9:**
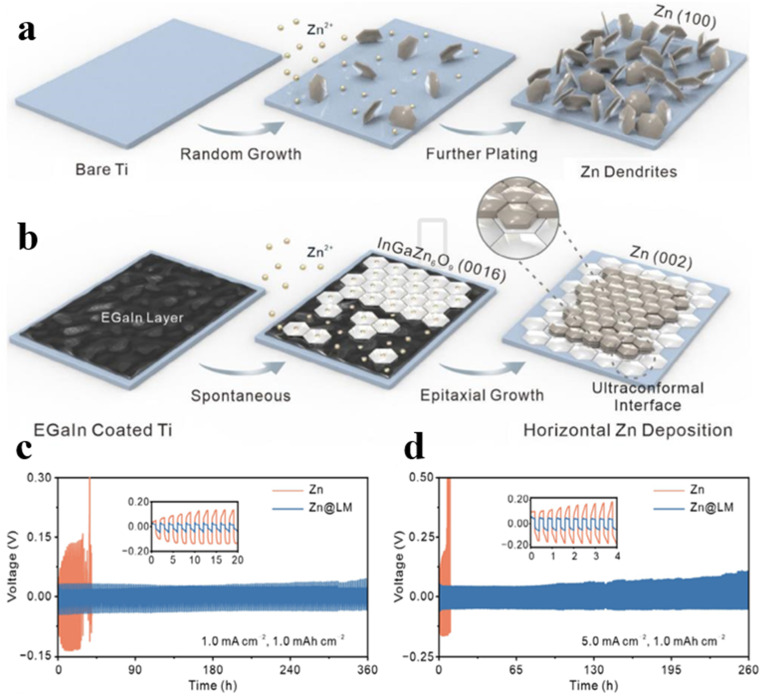
Scheme for Zn deposition process of (a) Ti and (b) LM@Ti anodes. Extending cycling performance of Zn and Zn@LM (c) at 1.0 mA cm^−2^ and 1.0 mA h cm^−2^, and (d) at 5.0 mA cm^−2^ and 1.0 mA h cm^−2^.^76^

The growth of dendrites stems from the irregular deposition of zinc, followed by the random stacking of different crystalline surfaces (Fig. 9a). Rational screening and design of epitaxial guided modification protocols are of great significance to achieve optimal zinc anode deposition and stripping, thereby improving the performance of the overall AZIBs. Zhang *et al.* developed a uniform liquid epitaxial interface of InGaZn_6_O_9_ by using spontaneous alloying of liquid gallium–indium alloy (EGaIn) and zinc.^76^ As the exposed (0016) facet of the liquid interface can effectively match the (002) crystal face of Zn, Zn grains are preferentially oriented along the (002) facet for uniformly horizontal deposition (Fig. 9b). The designed liquid interface with its superior wettability and fluidity, can effectively fill microscopic defects and inhomogeneities on the Zn surface, thus forming a homogeneous covering layer to inhibit dendrite growth. Additionally, the unique amorphous epitaxial interface offers a large effective surface area, increasing active sites and facilitating fast zinc ion deposition/exfoliation. In Zn@LM symmetric cells, the capacity remains at 80 mA h g^−1^ after 4400 cycles at 5 A g^−1^, significantly enhancing cycle life and energy density (Fig. 9c and d).

Presently, developing substrate materials with similar lattice structures to foster uniform zinc metal deposition has emerged as a burgeoning modification approach to enhance the stability of anode surface. The epitaxial modification can effectively control the method and conditions of metallic zinc deposition, making the metallic zinc deposition more uniform and with higher crystallinity, which helps to improve the battery performance and promote applications in the field of energy storage, as shown in Table 4.

**Table 4:** Comparison of anode materials based on epitaxial-oriented modification strategies

Anode material	Electrolyte	Voltage hysteresis	Cycling performance	Ref.
(101)-Zn	2 M ZnSO_4_	∼100 mV (4 mA cm^−2^)	5300 h (4 mA cm^−2^)	77
ZnF_2_@Zn-IL	1 M ZnSO_4_ + 0.1 M MMImI	24 mV (1 mA cm^−2^)	6500 h (1 mA cm^−2^)	78
(002)-textured Zn	2 M ZnSO_4_	∼45 mV (1 mA m^−2^)	3280 h (1 mA m^−2^)	79
FCOF@Zn	2 M ZnSO_4_	60 mV (5 mA cm^−2^)	1700 h (5 mA cm^−2^)	80
ZnSe	2 M ZnSO_4_	41 mV (1 mA cm^−2^)	1530 h (1 mA cm^−2^)	81
FAG@Zn	2 M ZnSO_4_	40 mV (1 mA cm^−2^)	4000 h (1 mA cm^−2^)	82
Zn(CH_3_COO)_2_	1 M Zn(Ac)_2_	∼40 mV (1 mA cm^−2^)	3000 h (1 mA cm^−2^)	83
Zn@Cu(100)	2 M ZnSO_4_	59 mV (1 mA cm^−2^)	550 h (1 mA cm^−2^)	84
MOF-E@Zn	2 M ZnSO_4_	∼90 mV (2 mA cm^−2^)	900 h (2 mA cm^−2^)	85
NGO@Zn	2 M ZnSO_4_	∼40 mV (1 mA cm^−2^)	1200 h (1 mA cm^−2^)	86

## 4. Summary and prospect

In summary, AZIBs are poised to become pivotal in the realm of energy storage to promote the advancement of clean energy and sustainable energy provision. However, the current zinc anode is subject to face long-term challenges like metal dendrite growth and other water-induced side reactions (*e.g.*, corrosion, passivation, and hydrogen evolution reactions) during battery operation. This review systematically summarized host design approaches of zinc substrates, including three-dimensional structural design, zincophilicity regulation, and epitaxial-oriented modification. Reasonable structural regulation can control interfacial mass transfer, uniform Zn nucleation and regulate crystal growth, thereby affecting the electrochemical performance of Zn anode. The 3D architecture expands the specific surface area, makes the current density uniform, and effectively reduces it to below the critical current density for dendrite growth. In addition, zincophilicity regulates Zn nucleation sites to ensure uniform charge distribution and provide homogeneous nucleation sites. Moreover, epitaxial modification can effectively control the dominant crystal plane of metal zinc deposition and induce more uniform and highly crystalline Zn deposition. Although the host-design optimization strategies have made significant progress in alleviating the challenges, more efforts should be devoted to the following aspects to promote their further development.

(1) Despite the development of the host design of zinc anodes, the detailed mechanisms of modification and the processes of anodic deposition at the atomic/molecular level are partially unclear and still need further exploration. In addition, due to the complexity of the aqueous system, the deposition/dissolution mechanisms of the Zn anode are still unclear and debatable. It is necessary to combine *in situ* dynamic characterization techniques with theoretical computational simulations to deeply reveal the complex mechanisms of anode failure in the current and near future. A full understanding of Zn deposition can help to break through conventional means and develop more efficient modification schemes.

(2) Based on the understanding of zinc electrodeposition, a series of effective descriptors based on crystal nucleation and growth theory can be further developed, and quantitative criteria for zinc anode host stability can be gradually established. The establishment of design criteria can effectively unify the quantification and comparison of the actual effects of various modification strategies and is more helpful in predicting and screening more suitable matrix structure or chemical composition regulation strategies.

(3) Host engineering modification can promote uniform zinc deposition to alleviate zinc dendrite formation, but it cannot completely suppress water-induced parasitic reactions, resulting in still unreliable stability of the anodes. The challenges of AZIBs concerning zinc anodes are mutually interconnected. Therefore, addressing these issues requires comprehensive consideration of various factors. For example, constructing an interfacial protection layer with a dense structure or strong molecular interaction on the anode surface can act as a physical barrier to prevent water/oxygen from diffusing to the electrode interface, thereby reducing the water/oxide content at the interface and alleviating the side reactions caused by free water molecules. Therefore, combining substrate modification with optimization strategies of other components (*e.g.*, electrolyte design, separator optimization) by utilizing their synergistic effects can simultaneously regulate the zinc deposition behavior and control the reactivity of water molecules, thereby further achieving higher stability and longer cyclic lifespan of the zinc anode.

(4) Suitable substrate materials should have high structural stability without compromising cost-effectiveness. Regarding the complex process and high cost of functional and structural regulation on the anode hosts, the development of highly economical materials and technologies is conducive to further industrialization. Compared to the currently-used Zn foil anode, the Zn power demonstrates notable benefits as a new type of anode owing to its large-scale adaptability. However, its higher activity and surface area induce more severe parasitic reactions. How to strike a balance between cost and stability through structural design is the key to the future industrialization of zinc anodes.

## Data availability

No primary research results, software or code have been included and no new data were generated or analysed as part of this review.

## Conflicts of interest

There are no conflicts to declare.
